# An Analysis of New York Data: Fluctuations in Hospital Capacity Are Driven by Variability in Elective Admissions and Discharge Activity

**DOI:** 10.7759/cureus.58404

**Published:** 2024-04-16

**Authors:** Samita M Heslin, Mark Henry, Eugene Litvak, Adam J Singer, Henry Thode, Peter Viccellio

**Affiliations:** 1 Emergency Medicine, Stony Brook University, Stony Brook, USA; 2 Healthcare Optimization, Institute for Healthcare Optimization, Newton, USA

**Keywords:** throughput, discharges, hospital admissions, crowding, boarding

## Abstract

Background

Hospital overcrowding compromises patient safety. The contribution of variability in admissions and discharges to overall hospital capacity needs to be quantified. This study describes the statewide day-to-day fluctuation in the volume of hospitalized patients, the variability and pattern of hospital admissions and discharges throughout the week, and the contribution of Emergency Department (ED) vs. elective (non-ED) admissions and discharges to the overall variability in the system across the week.

Methodology

This is a retrospective analysis of the New York State Statewide Planning and Research Cooperative System database, in which all New York healthcare facilities submit patient-level data monthly. The study period was from January 01 to December 31, 2015. Outcomes included total volumes of admissions and discharges and length of stay sorted by patient origin (ED vs. non-ED admits (elective)) and service type (medicine vs. surgery) by day of the week.

Results

We studied 1,692,090 hospital admissions. Admissions were highest on Mondays and Tuesdays and steadily decreased throughout the week. There was little variability in the ED admissions throughout the week. Surgical elective admissions had significant variability throughout the week, with higher admissions at the beginning of the week. There was a significant difference (p < 0.01) between admissions on weekdays vs. weekends. Discharges increased from Monday to Friday, with a dramatic drop on the weekends, for both ED and elective pathways. Systemwide, on Monday, hospitals were 21% above the mean volume, and on Fridays, hospitals were 32% below the mean volume.

Conclusions

Overall hospital capacity shows dramatic variability throughout the week, driven primarily by elective admissions and discharges from any source throughout the week. Because elective admissions are schedulable, hospitals can reduce variability by smoothing scheduling. Increased weekend discharges will also improve capacity.

## Introduction

Hospitals operating at full or over census is endemic and widespread, a problem that has existed for decades. This results in serious quality-of-care issues, such as increased errors, higher risk of mortality, increased length of stay (LOS), nurse burnout, and increased readmissions [1-6]. This is also a costly problem which is harmful to the financial health of the hospital. Lack of hospital capacity can lead to Emergency Department (ED) boarding of admitted patients which limits ED space, overwhelms staff, and undermines the principal function of the ED. The resulting lack of ED capacity results in delays in care, increased medical errors, and decreased satisfaction of patients and staff [7,8].

In the face of inadequate capacity, there has been a push to expand capacity, primarily by adding beds to hospitals unable to absorb the volume of patients in need of hospitalization. This study examines whether driving forces limiting total capacity are driven by volume per se or by the variability of that volume in day-to-day admissions and discharges. Furthermore, this study examines whether the variability is due to emergency admissions, which cannot be controlled, or elective (non-emergency) admissions, which by their nature are schedulable and controllable.

Previous studies have demonstrated variability in admission rates throughout the week, with fewer discharges occurring on weekends and increased LOS for patients discharged after the weekend [9-17]. However, these studies have notable limitations, such as focusing on small segments of medical/surgical admissions or specific diagnoses in one or a few hospitals. It remains unclear whether these variabilities exist at the diagnosis, hospital, or healthcare system level. To our knowledge, there have been no large-scale studies incorporating hundreds of hospitals to investigate variabilities in admission rates, discharges, and LOS across the week. This study aims to address this gap.

Goals of this investigation

This study’s objective was to describe the total statewide day-to-day volume of hospitalized patients, measure variability throughout the week in hospital admissions and discharges, examine the contribution to variability from ED vs. elective (non-ED) admissions and discharges in New York hospitals, and describe the magnitude of variability in the system across the week.

## Materials and methods

This study was approved by the Institutional Review Board at Stony Brook University (approval number: 867827). The New York State Statewide Planning and Research Cooperative System (SPARCS) database was the source of data for analysis. On a monthly basis, all Article 28 healthcare facilities (including freestanding facilities) must submit data to the SPARCS database, which collects patient-level data on admissions, diagnoses, discharges, LOS, and more. New York hospitals represent a great cross-section of US hospitals, with a range of small to large hospitals in a variety of locations, such as rural, suburban, and urban areas.

The study period was from January 01 to December 31, 2015. During the study period, there were a total of 1,994,180 adult hospital admissions in the SPARCS database. Patients categorized as pediatric, psychiatric, or obstetric were excluded from the study to focus primarily on adult medical and surgical admissions. Additionally, patients who left against medical advice were excluded from the study as these patients were discharged before their treatment was completed.

Total volumes of admissions and discharges were examined and this data was further sorted by patient origin (ED vs. non-ED) and service type (medicine vs. surgery). In this study, we denoted non-ED admissions as elective as they were not admitted through the emergency service. Admissions were studied by examining the average number of admissions that occurred per day of the week across all New York hospitals, along with the average number of admissions that occurred on weekdays and weekends. Data was expressed as counts and compared using the t-test using Stata (StataCorp LLC, College Station, TX, USA). Additionally, admissions were further analyzed by their source, distinguishing between those admitted through the ED and those directly admitted as electives for each day of the week. Moreover, admissions were examined based on whether they were admitted to a medical service or a surgical service for each day of the week. To further understand the variability of admissions per day of the week, the seven-day average number of admissions per day was calculated and compared to the average number of admissions per day of the week, with the percentage difference reported.

Similarly, for discharges, the average number of discharges per day of the week across all New York hospitals, as well as the average number of discharges on weekdays vs. weekends were studied and compared using a t-test. Discharges were further investigated by where they were originally admitted from, distinguishing between those that were admitted through the ED and those directly admitted as electives for each day of the week. Additionally, discharges were examined based on whether they were discharged from the medical or surgical service for each day of the week. The seven-day average number of discharges per day was compared to the average number of discharges for each day of the week with the percentage difference reported.

Variability per day of the week was further analyzed by calculating the mean number of admissions per day. This number was compared to the difference between the admissions minus discharges per day and the percentage change was reported. Additionally, the mean LOS by admission and discharge day of the week was studied for ED vs. elective origin and medicine vs. surgical admission.

## Results

The inclusion criteria were met by 1,692,090 hospital admissions; 1,096,050 (65%) patients were admitted from the ED and 596,040 (35%) patients were admitted from the non-ED pathways. It was assumed for the study that non-ED patients are elective admissions.

**Figure 1:**
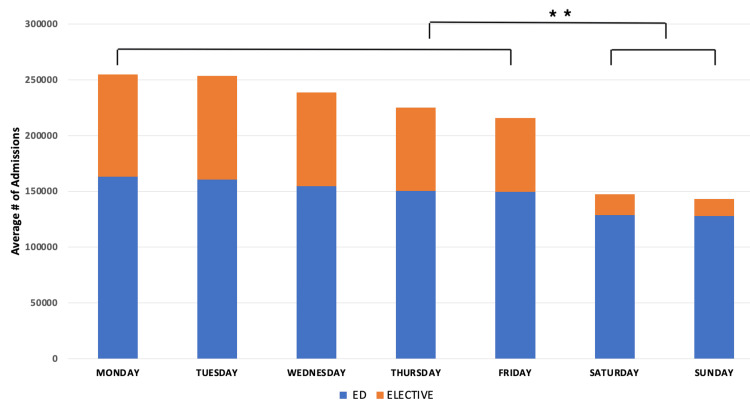
Admissions per day of the week from ED and elective pathways. P-values indicate statistical significance (**: p < 0.01). ED = Emergency Department

Admissions are highest on Mondays and Tuesdays and steadily decrease through the week for both elective and emergency admissions. For total admissions, ED admissions, and elective admissions, there was a significant difference (p < 0.01) between admissions on weekdays compared to weekends (Figure 1).

**Figure 2:**
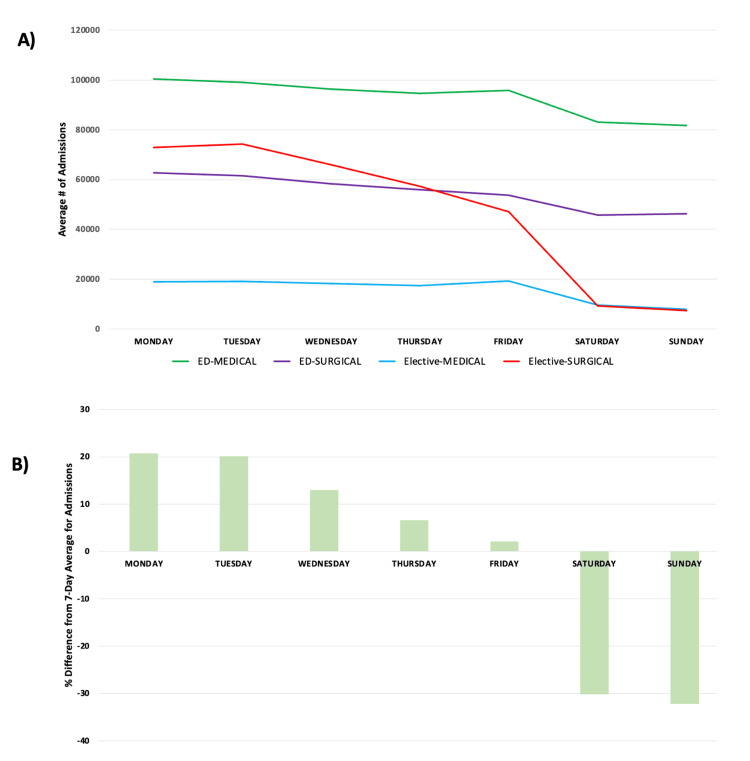
(A) Admissions per day of the week from the ED (medical vs. surgical) vs. elective (medical vs. surgical) pathways. (B) Percentage difference from the seven-day mean number of admissions by the day of the week. ED = Emergency Department

Admissions, when studied by ED vs elective and medical vs surgical pathways, show little variability in the number of ED patients who were admitted and went through medical or surgical pathways throughout the week. Similarly, elective medical admissions have little variability throughout the week. However, elective surgical admissions have significant variability throughout the week, with an increase in admissions at the beginning of the week and a decrease in admissions at the end of the week and on weekends (Figure 2a). When studying admissions by day of the week compared to the seven-day average number of admissions, the variability in elective surgical admissions is significant. On Sundays, there is a 32% decrease in admissions while on Mondays there is a 20% increase in admissions (Figure 2b). For the five-day week (Monday to Friday) the average number of admissions, elective admissions, varies from 13% above the mean to 19% below it.

**Figure 3:**
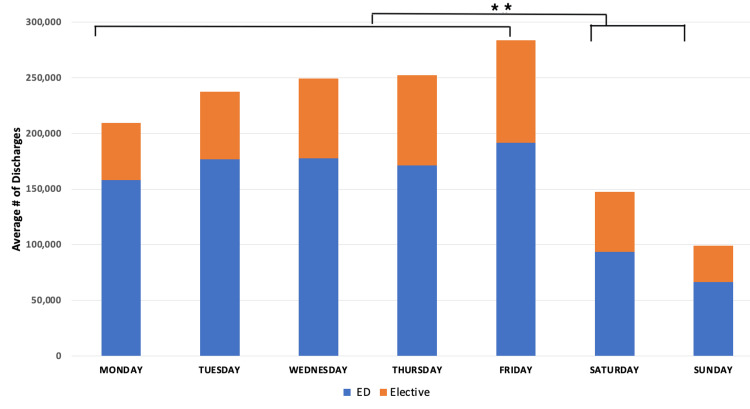
Discharges per day of the week from ED and elective pathways. P-values indicate statistical significance (**: p < 0.01). ED = Emergency Department

Discharges increase from Monday to Friday, with a dramatic drop on weekends. For total discharges, discharges of patients admitted through the ED, and discharges of patients admitted through the elective pathway, there was a significant difference (p < 0.01) between discharges on weekdays compared to weekends (Figure 3).

**Figure 4:**
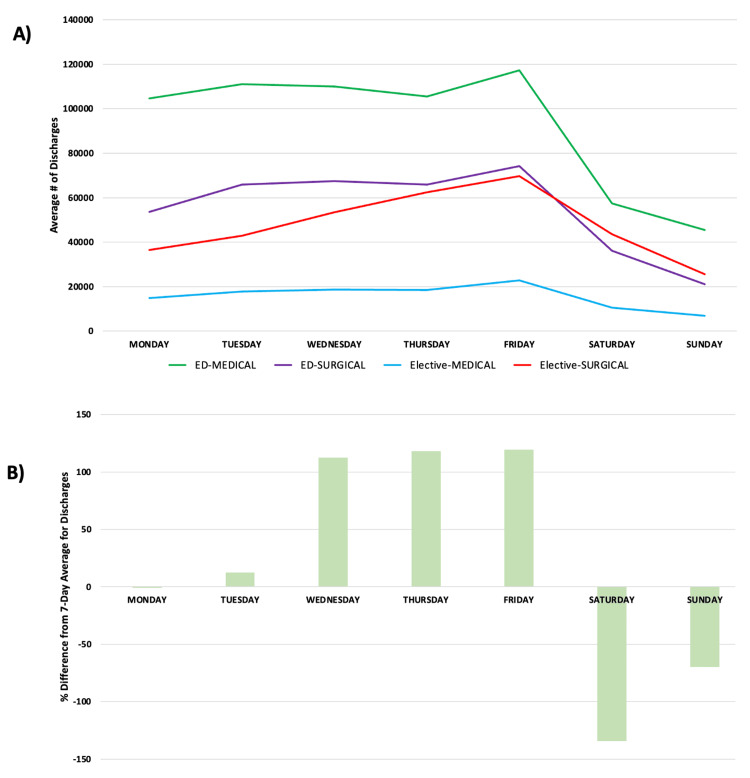
(A) Discharges per day of the week from the ED (medical vs. surgical) vs. elective (medical vs. surgical) pathways. (B) Percentage difference from the seven-day mean number of discharges by the day of the week. ED = Emergency Department

Discharges are highly variable, but relatively independent of source or type of admission. There are more discharges on the weekdays than on the weekends. Wednesday, Thursday, and Friday tend to have more discharges than Monday and Tuesday (Figure 4a). When studying discharges by day of the week compared to the seven-day average number of discharges, the variability was significant for all pathways, with a 134% decrease in discharges on Saturday and a 119% increase in discharges on Friday (Figure 4b).

**Figure 5:**
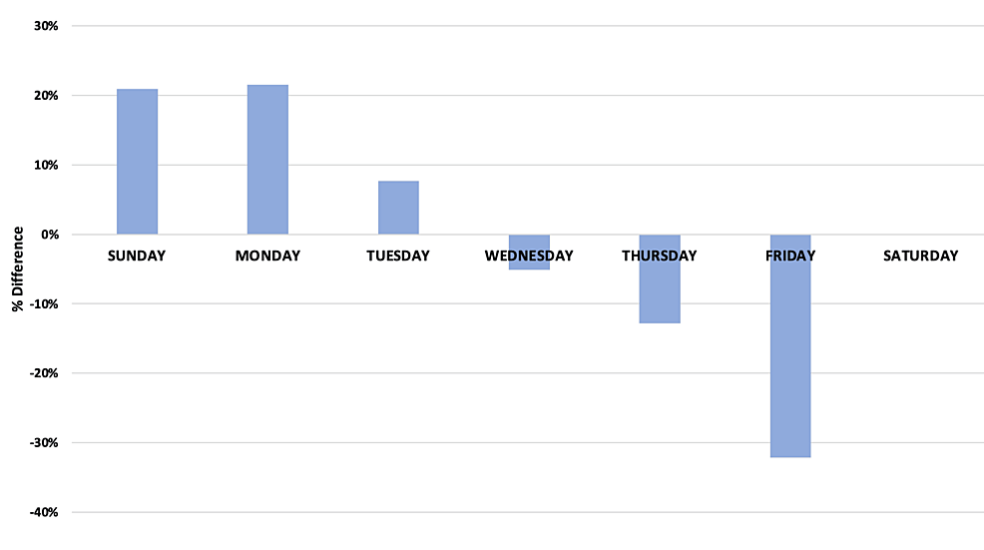
Difference between the seven-day average for admissions and the net admissions (admissions minus discharges) for each day of the week.

The variability in admissions is unimportant if offset by a complementary variability in discharges, i.e., if influx equals efflux. Overall hospital census, defined as admissions minus discharges, is significantly above the mean at the beginning of the week, and significantly below the mean at the end of the week, confirming substantial variability in the overall census. On Monday, hospitals were 21% above the mean volume, and on Fridays, hospitals were 32% below the mean volume (Figure 5). Applying this variability to an individual hospital, a 500-bed hospital would need 103 more beds to accommodate patients on Mondays and 160 fewer beds to accommodate patients on Fridays. The excess admissions early in the week is largely the combination of a simultaneous moderate increase in the number of emergency admissions and a substantial increase in the number of elective surgical admissions. ED admissions to medical services vary little throughout the week, but these patients are discharged at a much lower rate on weekends. Across all entry types (admissions and discharges) and services (ED and elective), LOS by day of discharge is higher on Monday and Tuesday and lower on Saturday and Sunday.

## Discussion

Previous studies have shown that elective or scheduled admissions drive the variability of day-to-day admissions, with most elective admissions occurring at the beginning of the week. Studies have also shown fewer discharges on the weekend and increased LOS for patients who are discharged after the weekend [9-17]. However, these studies were conducted in only one or a few hospitals, studied certain diagnoses, and did not include both medical and surgical cases. Our study analyzed admissions from all New York state hospitals, providing a broad cross-section of US hospitals. Our dataset encompasses hospitals of all sizes located in diverse settings, including rural, suburban, and urban areas. To our knowledge, this is the first and largest study of the variability of the hospital system as a whole. Our study agrees with previous smaller studies. Mondays and Tuesdays are the busiest days of the week in a hospital and are the times when crowding and boarding with all their adverse consequences are the greatest. Insofar as there are capacity limits in the system, our data suggests that limits are determined not by a lack of capacity but rather by a failure to manage capacity by more evenly distributing the workload across the week.

Although ED admissions can fluctuate daily, the nature of emergency admissions makes them unschedulable and unpredictable. The somewhat higher volume of ED admissions seen in the early part of the week may represent patients who waited until the beginning of the week to see their physician and were sent in when a serious concern was identified. Given the higher likelihood of a capacity problem, the ED may also be the path of admission for what otherwise would have been a direct non-ED admission due to a lack of beds. The degree to which these provide an explanation is unknown. Nonetheless, short of major events (epidemics, disasters, etc.) ED admissions are steady-state on weekdays. On the contrary, elective hospital admissions (e.g., most surgeries), which are schedulable and controllable, have a highly skewed pattern that drives wide fluctuations in the overall census. In current practice, the majority of elective admissions are scheduled early in the week, particularly surgical admissions. There are several reasons why elective surgeries are scheduled earlier in the week, including historical practices in surgical scheduling and workweek preferences [18,19]. Additionally, there may be decreased preoperative and postoperative resources over the weekends (e.g., ancillary services). These elective admissions create artificial variability in the hospital flow, resulting in significant fluctuations in overall hospital capacity. During peaks, elective admissions compete with ED admissions for inpatient floor and intensive care unit (ICU) beds, leading to ED boarding, hospital overcrowding, and diversions [9]. Unfortunately, this data demonstrates that Monday, the day with the highest number of ED admissions, coincides with the highest number of elective admissions.

Additionally, hospital discharges are crucial to open beds for newly admitted ED patients. As another source of significant variability, discharges are fewer during the weekends than the weekdays. Prior studies have shown that discharging patients earlier in the day and increasing discharges on weekends improve hospital capacity [20,21].

Some have described the hospital system as a five-day-a-week solution to a seven-day-a-week problem. Given the dramatic drop-off in elective admissions on Thursday and Friday, even a five-day week might represent an overstatement. This study demonstrates the significant variability in overall hospital capacity in New York State throughout the week, driven by completely mismatched rates of admission and discharge over the week. Overall, hospital admissions increased at the beginning of the week (Figures 1, 2), with far fewer admissions on weekends. These admissions are not matched by an equivalent number of discharges on Sunday, Monday, and Tuesday (Figure 5), thus driving capacity significantly above the mean. The main controllable driver for this variability is from non-ED admission sources, as well as widely variable discharge rates.

The mean LOS varies little by day of admission, but the mean LOS by day of discharge shows increased LOS for patients discharged on Monday compared to the weekend. The most variability in LOS by admission or discharge day is seen in elective admissions. The increased LOS demonstrated in patients discharged on Monday and Tuesday strongly suggests a spillover of patients who could potentially have been discharged over the weekend, which would have reduced capacity demand on Monday and Tuesday.

Smoothing of elective admissions has been shown to dramatically improve hospital capacity [22]. Smoothing elective surgeries (based on inpatient and ICU bed needs) across the week helps keep the daily number of resources constant, which helps with predicting staffing numbers (patient-per-nurse staffing ratios) and assists with planning bed assignments for patients. As many surgical patients need ICU and floor beds after surgeries, smoothing minimizes the competition for floor and ICU beds between elective and ED patients, which reduces ED boarding. To smooth elective surgeries, some surgeons who generally operate earlier in the week could move their surgeries to other days; thus, surgeons will not need to work more days, but rather on different days. By smoothing elective admissions, Cincinnati Children’s Hospital cut wait times by 30%, increased revenue by $137 million, and avoided a $100 million, 75-bed expansion [15,23].

Increasing patient discharges on the weekend can decrease ED boarding and decrease overall hospital LOS while increasing overall hospital capacity [21]. Increasing availability of patient services, such as physical therapy, on evenings and weekends can help prepare patients for a weekend discharge. When Montefiore Medical Center, which operates near 100% capacity, increased the number of weekend discharges by using throughput managers to assist with the discharge process, ED boarding decreased from an average of 30 patients to nearly zero in a year and a 30-bed inpatient unit was closed due to increased capacity [20].

The methods in this study are straightforward and could easily be applied to an individual hospital’s data to measure the degree of variability within a single institution. This could serve to instruct the institution to where their greatest opportunities are for capacity management and improvement.

Limitations

We used a large administrative database, with data submitted by hundreds of hospitals. Although the variability in overall activity statewide is striking, individual hospitals may have quite different experiences. Additionally, the data set is only from New York State; there may be some differences in other states based on hospital and patient characteristics, although the findings here are consistent with experience elsewhere [9-17]. As this study did not include pediatric, psychiatric, or obstetric patients, the findings are not generalizable to these populations. Furthermore, non-ED admissions were categorized as elective in this study, but there may be some elective admissions that cannot be scheduled. It is also possible that some elective-type cases may have been admitted through the ED, while some emergencies were directly admitted. While we anticipate these to be a small proportion relative to the dataset, we recommend that future studies investigate these aspects further.

## Conclusions

In this study of 1,692,090 hospital admissions in New York, we show that overall hospital capacity has significant variability through the week, driven primarily by variabilities in elective admissions and discharges from any source. There may be opportunities to reduce these problems in several ways. Because elective admissions are schedulable, hospitals may be able to reduce variability by smoothing scheduling. Further, increasing weekend discharges may improve capacity. Hospitals should evaluate ways to smooth elective admissions throughout the week while deploying resources to increase discharges on weekends.
